# EF24 inhibits tumor growth and metastasis via suppressing NF-kappaB dependent pathways in human cholangiocarcinoma

**DOI:** 10.1038/srep32167

**Published:** 2016-08-30

**Authors:** Da-long Yin, Ying-jian Liang, Tong-sen Zheng, Rui-peng Song, Jia-bei Wang, Bo-shi Sun, Shang-ha Pan, Lian-dong Qu, Jia-ren Liu, Hong-chi Jiang, Lian-xin Liu

**Affiliations:** 1 Department of General Surgery, the First Affiliated Hospital of Harbin Medical University; Key Laboratory of Hepatosplenic Surgery, Ministry of Education. No23, Youzheng Street, Nangang District, Harbin, Heilongjiang Province, 150001, P.R.China; 2 Department of Pharmacology, The State-Province Key Laboratories of Biomedicine- Pharmaceutics of China, Harbin Medical University, Harbin, Heilongjiang 150081, PR China; 3 National Key Laboratory of Veterinary Biotechnology, Harbin Veterinary Research Institute of Chinese Academy of Agricultural Sciences, Harbin, P.R. China; 4 Department of Anaesthesia, Harvard Medical School, Boston, MA, USA

## Abstract

A synthetic monoketone analog of curcumin, termed 3, 5-bis (2-flurobenzylidene) piperidin-4-one (EF24), has been reported to inhibit the growth of a variety of cancer cells both *in vitro* and *in vivo*. However, whether EF24 has anticancer effects on cholangiocarcinoma (CCA) cells and the mechanisms remain to be investigated. The aim of our study was to evaluate the molecular mechanisms underlying the anticancer effects of EF24 on CCA tumor growth and metastasis. Cell proliferation, apoptosis, migration, invasion, tumorigenesis and metastasis were examined. EF24 exhibited time- and dose-dependent inhibitory effects on HuCCT-1, TFK-1 and HuH28 human CCA cell lines. EF24 inhibited CCA cell proliferation, migration, and induced G2/M phase arrest. EF24 induced cell apoptosis along with negative regulation of NF-κB- X-linked inhibitor of apoptosis protein (XIAP) signaling pathway. XIAP inhibition by lentivirus mediated RNA interference enhanced EF24-induced apoptosis, while XIAP overexpression reduced it in CCA cells. *In vivo*, EF24 significantly suppressed the growth of CCA tumor xenografts and tumor metastasis while displaying low toxicity levels. Our findings indicate that EF24 is a potent antitumor agent that inhibits tumor growth and metastasis by inhibiting NF-κB dependent signaling pathways. EF24 may represent a novel approach for CCA treatment.

Cholangiocarcinoma (CCA) is the second most common hepatobiliary malignancy, and its incidence has increased in recent years, with approximately 5,000 new cases in the United States per year^1^,^2^. Current therapeutic options for CCA are limited, and the overall prognoses for patients with CCA remain dismal^3^,^4^,^5^,^6^. Although surgical resection is the primary therapeutic option for CCA, most patients have advanced disease at the time of diagnosis and are not suitable candidates for surgery^7^,^8^,^9^. Although advanced CCA responds to chemotherapeutic agents such as gemcitabine, cispaltin and sorafenib, there is no established standard regimen or protocol^10^,^11^,^12^. Therefore, further studies are clearly necessary for the development of novel therapeutic options for patients with CCA.

Curcumin (diferuloylmethane) is a yellow component of all curry powders and pastes and has been widely used in Eastern traditional medicine. Recently, curcumin has attracted great attention as a possible novel anticancer agent due to its antiproliferative and antiangiogenic properties, while displaying minimal toxicity^13^,^14^. However, the molecular mechanism of action associated with curcumin remains a subject of debate. Prakobwong and colleagues reported that curcumin stimulated antiproliferative and apoptotic effects in CCA through multiple cell signaling pathways, including the inhibition of cell survival protein expression such as Bcl-2, Bcl-xL, X-linked inhibitor of apoptosis protein, c-FLIP, cellular inhibitor of apoptosis protein (cIAP)-1, cIAP-2 and surviving^15^. Unfortunately, curcumin’s low bioavailability hinders further clinical development^16^. Recently, a synthetic monoketone compound termed 3, 5-bis (2-flurobenzylidene) piperidin-4-one (EF24) has been shown to have increased anticancer activity with a safety profile similar to curcumin^17^,^18^,^19^. Studies in various cancer cell types have suggested that EF24 impairs cell growth by inducing G2/M arrest followed by induction of apoptosis, which is accompanied by caspase-3 activation. In our previous study^20^,^21^, we showed EF24 induced significant apoptosis and G2/M phase cell cycle arrest mediated by the downregulation of NF-κB expression in different hepatocellular carcinoma (HCC) cell lines, indicating that EF24 might be a therapeutic option for HCC. However, the effects of EF24 on human CCA remain to be elucidated. In this study, we sought to evaluate the underlying molecular mechanisms regulating the effects of EF24 on tumor growth and metastasis using human CCA cell lines (HuCCT-1, TFK-1 and HuH28) and tumor xenografts in order to explore the potential of EF24 as a new treatment option for CCA.

## Results

### EF24 inhibits CCA cell proliferation and induces G2/M cell cycle arrest

**Figure 1:**
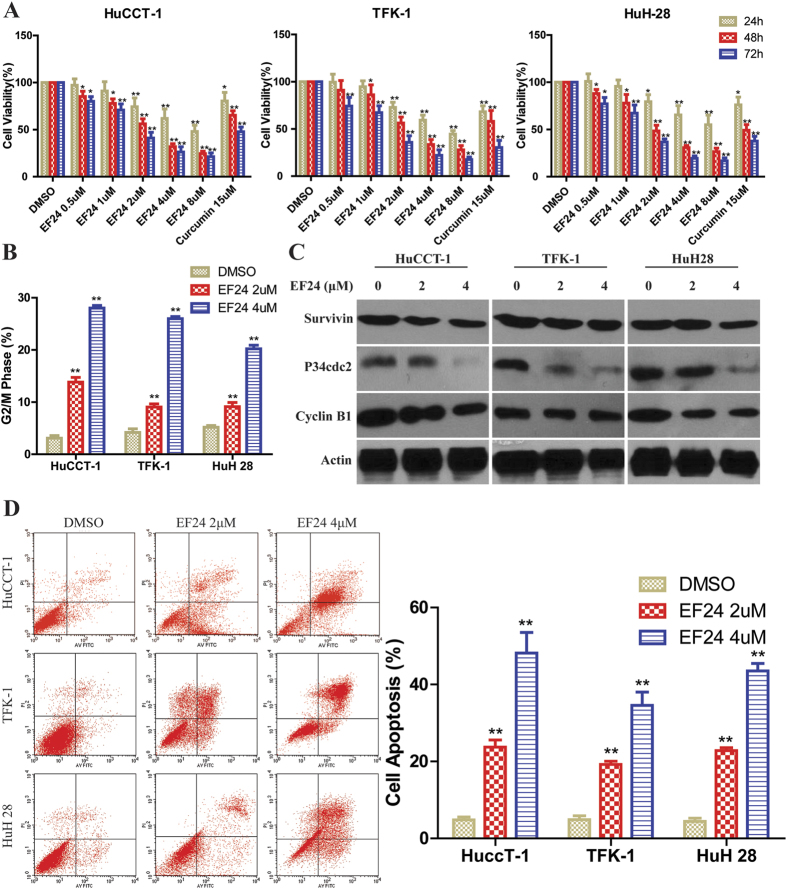
EF24 inhibited CCA cell proliferation, induced cell cycle arrest and apoptosis. **(A)** Cells were incubated with increasing doses of EF24 (0.5–8 μmol/L) for 24, 48 and 72 h periods to analyze cell proliferation using the cell counting kit-8 assay. **(B**) EF24 treatment led to increased number of cells in the G2/M phase after 48 h compared with untreated controls. **(C)** Western blot analysis showed that the cell cycle related proteins P34^cdc2^, cyclin B1 and survivin expression were changed by EF24. **(D)** Three CCA cell lines were incubated with EF24 (2 or 4 μmol/L) for 48 h and analyzed for apoptosis by Annexin V-FITC staining. EF24 treatment increased number of apoptotic cells in a dose-dependent manner compared with the untreated control.EF24 treated *versus* untreated control. *P < 0.05; **P < 0.001.

We first determined the effects of EF24 on cell proliferation in three CCA cell lines. All cells were treated with various concentrations of EF24 and 15 μmol/L of curcumin for 24, 48 and 72 h. As shown in (Fig. 1A), EF24 significantly suppressed the proliferation of human HuCCT-1, TFK-1 and HuH28 cells in a dose- and time- dependent manner, with the most marked results observed in HuCCT-1 cells. More importantly, the effects were observed at a dose of 2 μmol/L, a dose at which curcumin had no significant effects. Dose-and time- response studies revealed that the IC50 values of EF24 were in the range of 1.1 to 2.0 μmol/L for various cell lines, while the IC50 value for curcumin ranged from 15 to 20 μmol/L under the same treatment conditions, which may explain more potent antiproliferative properties of EF24 than curcumin in CCA cells. As shown in (Supplementary Fig. S1A and Fig. 1B), treatment with EF24 induced G2/M phase cell cycle arrest in a dose-dependent manner in CCA cells. Western blot results showed the changes of the expression of G2/M cell cycle regulating factors, such as survivin, cyclin B1 and cdc2 (Fig. 1C). These data suggested that the inhibition of cell proliferation by EF24 is associated with the induction of G2/M phase arrest, which may result from decreased survivin, cdc2 and cyclinB1 expression.

### EF24 induces CCA cell apoptosis

Arrest of cell cycle progression in tumor cells is usually associated with concomitant activation of cell death pathways^21^,^22^,^23^,^24^,^25^,^26^. Here, the flow cytometery and confocal microscopy were employed to determine whether CCA growth inhibition mediated by EF24 resulted from apoptosis. Results indicated that CCA cells showed a dose-dependent apoptosis after EF24 treatment, including early and late apoptosis (Fig. 1D and Supplementary Fig. S1B).

### EF24 suppresses the NF-κB/XIAP pathway in CCA cells

**Figure 2:**
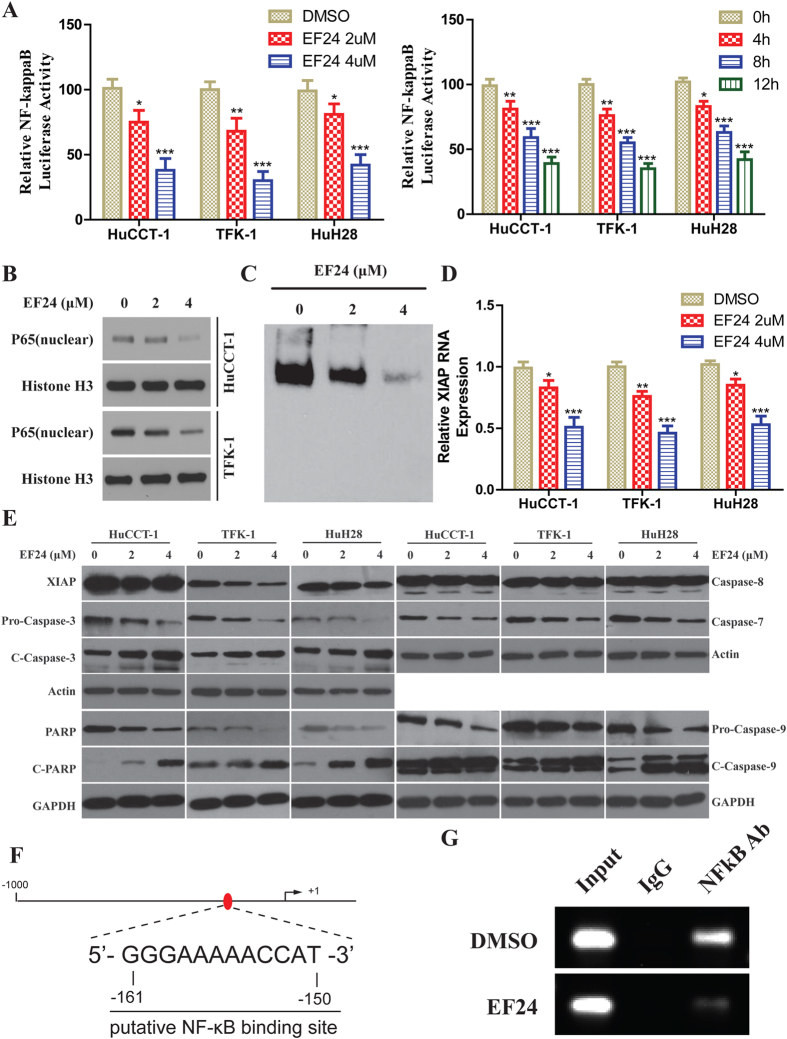
Inhibition of NF-κB Pathway of CCA Cells by EF24. (**A**) (Left-panel) Cells were transfected with pNF-κB-Luc. Transfected cells were treated with EF24 for 12 h and analyzed with an assay kit; (Right-panel) Transfected cells were treated with 2 μM EF24 for 4, 8, and 12 h. Relative luciferase activities are normalized to the untreated (control) samples. **(B)** Cellular nuclear proteins were extracted and underwent Western blot analysis. **(C)** DNA-binding affinity was determined in treated cells by EMSA using an assay kit in HuCCT-1 cells after EF24 treatment. **(D)** The real-time PCR was used to analyze the XIAP mRNA levels followed EF24 treatment. All assays were conducted in triplicate. **(E)** Western blot analysis showed that EF24 reduced XIAP expression and activated caspase-9, caspase-3, caspase-7 and PARP. **(F)** Schematic diagram of the XIAP proximal promoter. The nucleotide positions and sequences of the putative NF-κB binding site located −161/−150bp upstream of the transcriptional start site in the XIAP promoter. **(G)** ChIP assays in samples produced from HuCCT-1 treated by DMSO and EF24. PCR was performed with primers specific for NF-κB binding site. Data are presented as the mean ± SD with *p < 0.05, **p < 0.01, ***p < 0.001.

To investigate the inhibitory effects of EF24 on NF-κB function, NF-κB transcriptional activity was investigated by promoter assay. Suppression of NF-κB transcriptional activity was observed in EF24 treated CCA cells in dose- and time-dependent manners as shown in (Fig. 2A). Western blot analysis of nuclear p65 suggested the reduction of NF-κB DNA binding and transcriptional activity were caused by reduced active p65 protein (Fig. 2B).To further clarify the NF-κB inhibitory effects of EF24, NF-κB-DNA binding capability was determined by EMSA. Inhibition of NF- κB-DNA binding could be observed in a dose-dependent manner (Fig. 2C) in HuCCT-1 cells. As reported, XIAP is a key NF*-*κB downstream target protein inhibiting apoptosis, which usually over-expresses at multiple stages of CCA carcinogenesis^21^,^22^,^23^,^24^. First, RT-PCR result showed a significant decrease in XIAP mRNA level (Fig. 2D). Next, Western blot analysis showed a significant decrease in XIAP, and activated caspase-3 in CCA cells after EF24 treatment. The analysis demonstrated activation of the caspase cascade, including PARP, caspase-9 and -3. Nevertheless, EF24 did not activate caspase-8 in CCA cells (Fig. 2E). We further elucidated the mechanism by which XIAP expression was regulated in CCA. Bioinformatics analysis demonstrates the presence of one candidate NF-κB binding site in the snai1 promoter located −161/−150 bp upstream of the transcriptional start site (Fig. 2F). ChIP experiments were thus performed with HuCCT-1 cell samples treated by DMSO and EF24 to determine whether NF-κB binds to this site. As shown in (Fig. 2G), NF-κB bound to the site in the XIAP promoter, the activity of binding to XIAP promoter decreased after EF24 treatment.

### XIAP shRNA increases the apoptosis of CCA cells induced by EF-24

**Figure 3:**
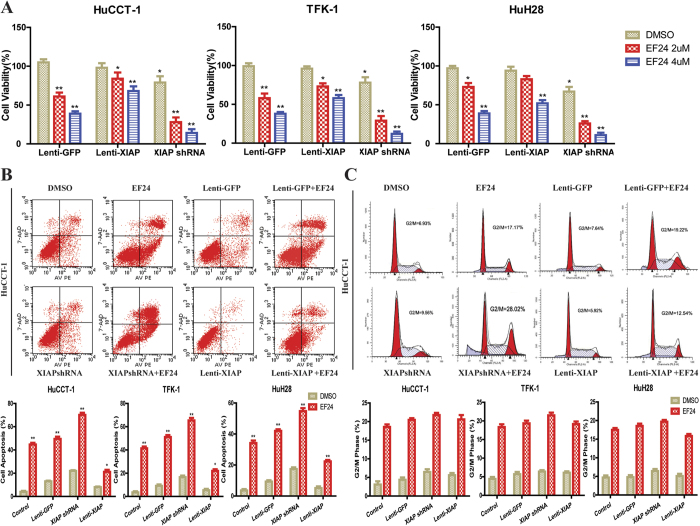
XIAP shRNA enhanced the CCA cell growth inhibition and apoptosis induction by EF24. **(A)** HuCCT-1, TFK-1 and HuH28 CCA cells transfected with Lenti-GFP, Lenti-shRNA-XIAP and Lenti-XIAP were incubated with EF24 for 48 h to analyze cell viability using CCK8 assay. **(B)** XIAP suppression enhanced EF24-mediated CCA cell apoptosis, and overexpression of XIAP markedly reduced apoptosis. **(C)** Suppression or overexpression of XIAP did not affect the CCA cell G2/M cell cycle arrest induced by EF24 treatment. The assay was conducted in triplicate. *P < 0.05, **P < 0.001, EF24 treated *versus* untreated control.

As mentioned, EF24 induced CCA cell apoptosis accompanied by XIAP decrease and subsequently activating the caspase cascade. We further examined the exact role of XIAP in EF24-mediated CCA cell apoptosis. We introduced Lenti-XIAP and Lenti-shRNA targeting XIAP into CCA cells. XIAP expression was remarkable decreased by Lenti-shRNA1 and moderately reduced by other three shRNAs compared to the control shRNA(Supplementary Fig. S3A). The downregulation of XIAP protein was confirmed by Western blotting (Supplementary Fig. S3B). The XIAP-knockdown cells were used to test the effects of reduced XIAP levels on cell sensitivity to EF24. As shown in (Fig. 3A), XIAP suppression by shRNA enhanced sensitivity of CCA cells to EF24, while XIAP overexpression reduced the sensitivity of CCA cells to EF24, indicating a coordinating role between XIAP suppression and EF24. Importantly, the suppression of XIAP in CCA cells enhanced apoptosis induced by EF24 (Fig. 3B and Supplementary Fig. S2), characterized by enhanced activation of caspase-9 and -3 (Supplementary Fig. S3C). On the other hand, XIAP overexpression markedly reduced the apoptosis mediated by EF24. In addition, XIAP suppression resulted in increased activation of caspase-7 and PARP levels, but not caspase-8 (Supplementary Fig. S3C). However, the suppression or overexpression of XIAP did not affect CCA cell cycle arrest followed by EF24 treatment compared with those cells without XIAP suppression or overexpression (Fig. 3C and Supplementary Fig. S4).

### EF24 inhibits CCA cell migration and invasion by suppressing epithelial-mesenchymal transition (EMT) and regulating cytoskeletal structure and focal adhesion formation

**Figure 4:**
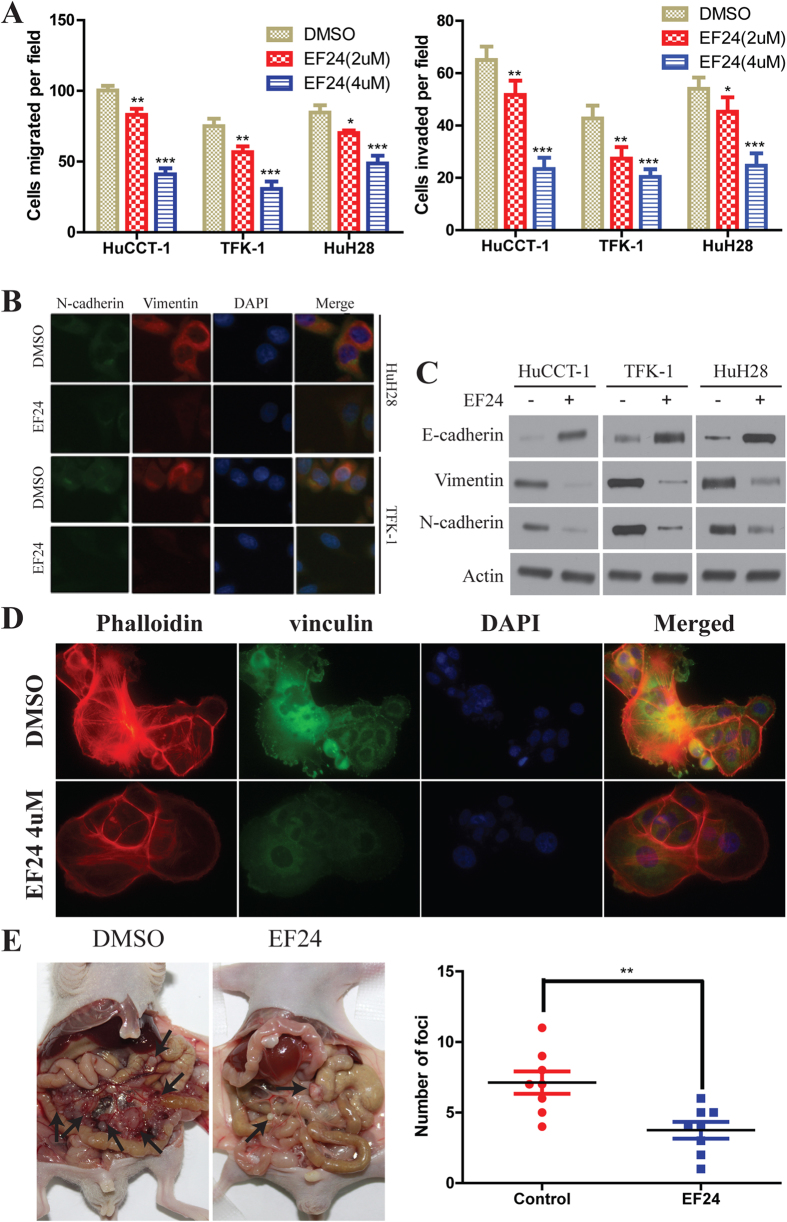
EF24 inhibits CCA tumor metastasis. **(A)** Migration (Left-panel) and invasion (Right-panel) assays of the indicated CCA cell lines. Cells that migrated and invaded to the bottom of the chamber were counted in three fields under × 20 magnifications. **(B)** Single and merged images were taken to show immunofluorescence staining of N-cadherin (green) and vimentin (red) accompanied by the cell nucleus (blue) stained by DAPI. **(C)** 24 hours after treatment, immunoblotting showed EF24 (2 μM) increased the expression of E-cadherin and decreased the expression of N-cadherin and vimentin in CCA cells. **(D)** EF24 inhibited HuCCT-1 cells stress fiber and focal adhesion formation. HuCCT-1 cells were treated with DMSO or EF24. Cells were stained with rhodamine-phalloidin and vinculin antibody (Green = vinculin, red = F-actin). **(E)** The multiple tumor masses formed by the HuCCT-1 cells in the control group were much less than that by HuCCT-1 cells in the EF24 treated group. All data were the means ± SD of three separate experiments. *P < 0.05, **P < 0.01, ***P < 0.001.

We also examined the ability of EF24 to modify cell migration and invasion using the transwell migration and matrigel-based invasion assays. In the transwell migration assay, the results indicated that EF24 significantly inhibited the migration of the CCA cells compared with the vehicles. The invasion assay results also indicated that EF24 significantly reduced the invasion of CCA cells through matrigel membrane (Fig. 4A). These results indicate that EF24 suppresses the motility of CCA cells. Given that EF24 inhibits CCA metastasis, we investigated the effect of EF24 on EMT, a critical event in tumor invasion. Immunofluorescence assay detected lower expression of N-cadherin and vimentin in CCA cells which treated with EF24 (Fig. 4B). The epithelial marker such as E-cadherin was higher in the EF24 group than that in the DMSO group, whereas the mesenchymal markers such as N-cadherin and vimentin were decreased (Fig. 4C). We further examined the exact role of XIAP in EF24-mediated the change of CCA cell EMT markers. We also introduced Lenti-XIAP and Lenti-shRNA targeting XIAP into CCA cells. Our data showed that the inhibition of XIAP expression partially enhanced the increase of E-cadherin expression induced by EF24, whereas inhibition of XIAP expression enhanced the decrease of N-cadherin and Vimentin expression induced by EF24 (Supplementary Fig. S5A). In contrast, overexpression of XIAP partially inhibited the decrease in E-cadherin expression induced by EF24, whereas overexpression of XIAP inhibited the increase of N-cadherin and Vimentin expression induced by EF24 (Supplementary Fig. S5A). Since cytoskeletal organization and focal adhesion maturation are essential for cell migration^27^, we next analyzed the dynamics of actin cytoskeleton and focal adhesions. HuCCT-1 cells were equipped with stress fibers with thick actin bundles traversing the cell bodies. EF24 exposure abrogated the stress fiber structure (Fig. 4D). Quantitative analysis unraveled that EF24 exposure decreased the size and number of focal adhesion sites (Supplementary Fig. S5B,C). These observations demonstrate that EF24 affects the organization of the actin filament network and the maturation of focal adhesions. The effects of EF24 on the metastatic phenotype of CCA were also examined *in vivo* by implanting CCA cells into the peritoneal cavity of nude mice. A necropsy after 5 weeks revealed that the control cells extensively colonized the visceral organs and formed multiple metastatic nodules (Fig. 4E), while the number of metastatic nodules was reduced in mice of EF24 treated group.

### EF24 inhibits tumor growth *in vivo*

**Figure 5:**
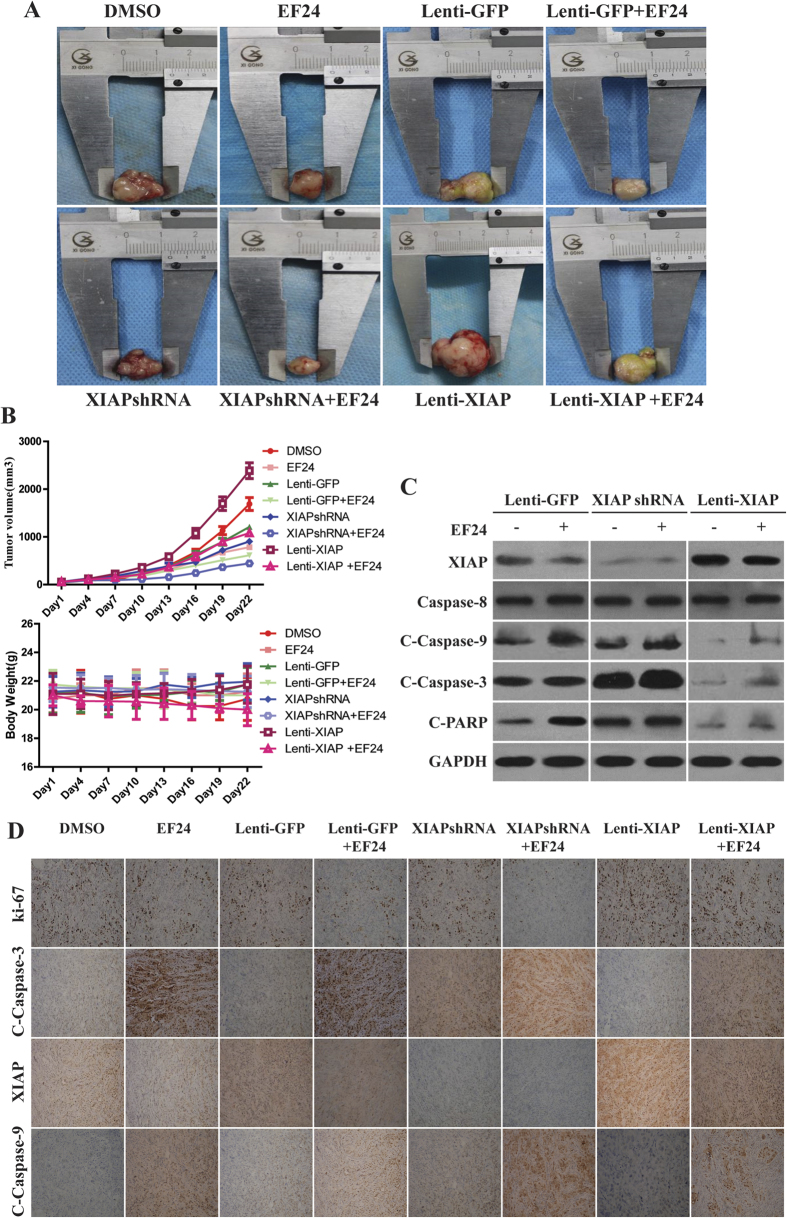
EF24 inhibits CCA tumor growth *in vivo*. **(A**) HuCCT-1cells were injected to the flanks of nude mice and palpable tumors were allowed to develop for 7 d. Subsequently, EF24 was injected daily i.p. for up to 21 d. On day 22, tumors were excised and subjected to further analyses. Representative tumors of each group were showed. **(B)**Tumor size and mice body weight were measured every three days. There was a significant reduction in relative tumor volume from EF24-treated animals when compared with untreated controls. **(C)** Western blot showed that the suppression of XIAP in CCA xenografts enhanced apoptosis induced by EF24, followed by enhanced activation of caspase-9 and -3, whereas caspase-8 expression was not affected by XIAP expression increase or decrease. **(D**) Representative results of Ki-67, XIAP, cleaved caspase-9 and -3 staining in the treated and the control tumors (×200).

To evaluate the effects of EF24 on tumor growth *in vivo*, we examined the ability of EF24 to suppress tumor growth *in vivo*. HuCCT-1 CCA xenografts were established and grew to a size of 100 mm^3^, after which EF24 was given i.p. daily for 3 weeks. In general, the tumors in control group grew continuously during the experimental period whereas the tumor growth in the EF24-treated mice was significantly suppressed. The growth suppression induced by EF24 was more significant in xenografts derived from CCA cells transfected with XIAP shRNA (Fig. 5A). The average weight of tumors from the EF24-treated animals was 377 mm^3^, whereas those from the control group weighed 2,565 mm^3^. To evaluate whether EF24 administration affects normal physiology, we treated non–tumor-bearing mice weekly, at exactly the same 20 mg/kg/d body weight dose, for 3 weeks. No apparent toxicity advents were observed in EF24-treated animals. There were no significant changes in body weight in the animals (Fig. 5B). In case of apoptosis, as shown in the representative photographs, tumor xenografts from the EF24-treated groups showed a marked increase in TUNEL-positive cells compared with the control group (Supplementary Fig. S6). The western blot results revealed that EF24 decreased the expression of XIAP and increased the expression of cleaved caspase-9 and caspase-3 (Fig. 5C).The positive tumor cells for Ki-67, a cell proliferation marker, were substantially less in tumors treated with EF24, compared with control tumors (Fig. 5D).

## Discussion

Cholangiocarcinoma (CCA) is the second most common hepatobiliary malignancy, and has emerged as one of the leading causes of cancer-related deaths in the Western world. The significant morbidity of surgery and low efficacy of chemotherapy for CCA has led to exploration for less toxic alternative therapies. A body of evidence indicates that regulations of aberrant NF-kB and the signaling pathways that control its activity are involved in cancer development and progression, as well as in drug resistance, especially during chemotherapy and radiotherapy^28^,^29^,^30^. Seubwai *et al.* have demonstrated that all NF-kB subunits were over-expressed in CCA patient tissues. This finding also suggests NF-kB as an attractive molecular target for CCA therapy^31^. Many studies have shown that EF24, a novel curcumin analogue, suppresses the proliferation of a variety of tumor cells, including breast, gastrointestinal, ovarian cancer and hepatocellular carcinoma with potency much higher than that of curcumin^17^,^18^,^19^,^20^,^21^. Our results indicated that EF24 possesses great potential as a therapeutic agent for CCA. Consistent with previous reports, EF24 exhibited IC50 values which are 10 to 20 times lower than that of curcumin in CCA cell lines^25^,^26^,^32^,^33^,^34^. The data presented in this article shows that EF24 selectively inhibits the proliferation and migration of CCA cancer cells, suppresses the CCA tumor growth and metastasis *in vivo*, inhibits NF-κB dependent pathways, induces cell cycle arrest and apoptosis.

Adams *et al.* showed that EF24 induced G2/M phase cell cycle arrest in both human breast cancer cells and human prostate cancer cells, and Selvendiran *et al.*^34^ reported that the inhibitory effect of EF24 on cisplatin-resistant human ovarian cancer cell proliferation is associated with G2/M phase cell cycle arrest. We have demonstrated here that EF24 could also induce G2/M cell cycle arrest in all the three selected CCA cell lines. Besides, the observation of the cell cycle related protein levels showed that, after EF24 treatment, the expression of survivin and Cyclin B1 was markedly decreased. Cyclin B1/cdc2 kinase plays a critical role as M-phase promoting factor in the G2/M transition^35^,^36^,^37^. Our results suggested that EF24 induced CCA cell cycle arrest, which may decrease Survivin expression, subsequently suppress the cdc/cyclin B1 kinase activation.

Previous studies have suggested that EF24 could activate caspase-3, and induce cleavage of PARP, leading to cell death/apoptosis^18^,^32^,^34^. In this study, we showed that caspase activation was involved in EF24-induced CCA cell apoptosis. The analysis demonstrated activation of the caspase cascade, including caspase-9, -3, -7, and PARP after EF24 treatment. Interestingly, our results showed the inhibition of NF-κB transcriptional activity by EF24, which was consistent with our previous study in HCC^20^,^21^. This mechanism was supported by the reductions of NF-κB-DNA binding and levels of active p65 proteins in CCA cells after treatment with EF24. First reported in 1997, XIAP is a key NF-κB downstream protein that inhibits apoptosis and promotes chemotherapy resistance by blocking caspase-9 and-3 activation^38^,^39^,^40^. The activation of caspase-9 and -3 with the EF24 treatment in this study prompted us to examine the expression level of a possible upstream regulation gene XIAP. Consistent with our previous findings, EF24 markedly decreased XIAP mRNA and protein levels. We firstly found XIAP was transcribed directly by NF-κB in CCA, and EF24 decreased NF-κB bind to XIAP promoter, and then suppressed transcription of XIAP. To further examine the role of XIAP expression on cell proliferation and apoptosis, lentivirus-mediated XIAP knockdown and overexpression were employed. The suppression of XIAP in CCA cells and enhanced apoptosis induced by EF24, may be caused by enhanced activation of caspase-9 and -3. Interestingly, similar results were observed both *in vivo* and *in vitro*. The data here was inconsistent with the results of Selvendiran^34^ which suggested that EF24 induced cell apoptosis by activating caspase-8,-9 and -3. The contradictory results may be caused by the activation of different mechanisms by EF24 treatment in various cancer cell types^41^,^42^. For the *in vivo* studies, we found there was a significant reduction in relative tumor size and volume in EF24-treated animals compared to untreated controls. In addition, the suppression of proliferation by EF24 was confirmed by decreased Ki-67 immunostaining. Increased numbers of apoptotic cells and activated protein levels of the apoptosis–related genes cleaved caspase-9 and cleaved caspase -3 were accompanied by decreased XIAP expression in the EF24-treated animals.

In summary, we observed marked suppression of tumor growth and metastasis via suppressing NF-κB/XIAP dependent pathways by EF24 both in CCA cells and nude mice. To the best of our knowledge, we are the first to demonstrate that the synthetic curcumin analog EF24 possesses anticancer effects on human CCA both *in vitro* and *in vivo*. EF24 has greater biological activity and bioavailability than curcumin, but does not increase toxicity. EF24 seems to have multiple molecular targets and its promising potency in CCA cell lines and xenograft tumors renders it a strong candidate for therapeutic applications in CCA as well as other cancers.

## Materials and Methods

### Cells and reagents

Curcumin and EF24 were purchased from Sigma-Aldrich (St. Louis, MO) and dissolved in DMSO. Human HuCCT-1, TFK-1 and HuH28 CCA cells lines (kindly provided by Cancer Cell Repository, Tohoku University, Japan) were incubated in RPMI 1640 or α-MEM (Gibco) containing 10% heat-inactivated fetal bovine serum (FBS) (Sigma-Aldrich) supplemented with 1% penicillin-streptomycin solution (Gibco) at 37 °C in a humidified atmosphere of 5% CO_2_.

### Cell viability assays

Cell viability was measured using the Cell Counting Kit-8 (CCK-8, Dojindo Molecular Technologies, Japan), according to the manufacturer’s instructions.

### Migration and invasion assays

Human HuCCT-1, TFK-1 and HuH28 CCA cells were treated by EF24 (2 μmol/L or 4 μmol/L) for 24 h, and then the transwell migration and invasion assays were performed in the BD Falcon 24-multiwell insert system (BD Biosciences, San Jose, CA, USA) following the manufacturers’ instructions.

### Immunofluorescence

Briefly, cells were seeded on coverslips and treated 2 μmol/L for 24 h, and then cells were fixed with 4% (w/v) paraformaldehyde (Sigma) for 10 min and permeabilized with 0.1% (v/v) Triton X-100 for 5 min at room temperature. The cells were then incubated overnight with anti-N-cadherin, anti-vimentin or anti-vinculin (Supplementary Table 1) at 4 °C, followed by incubation with fluorescent secondary antibody (invitrogen) for 1 hour at room temperature. After final washes with PBS, the coverslips were mounted using an anti-fade mounting solution containing 4′,6-diamidino-2-phenylindole (DAPI; Vector lab, Burlingame, CA) and images were examined and captured. To image actin filaments, rhodamine-phalloidin (1:100; Molecular Probes, New York, US) was incubated with cells. Finally, fluorescent staining was visualized with Nikon Eclipse Ni-E upright fluorescent microscope (Nikon, Japan.) with 40× magnification. Colocalization of vinculin and actin was processed by Image J. To analyze the size and number of vinculin-containingfocal adhesions, images were background subtracted before thresholding and segmentation were conducted to detect the edges of focal adhesions. Then, the mean size (in pixels) and number of focal adhesions in each cell were calculated.

### Luciferase reporter assay for NF-κB transcriptional activity

pNF-κB-luciferase plasmid or control luciferase plasmid was constructed, and luciferase activity assays were performed as described^43^. Cells were seeded in triplicates in 6 well plates and allowed to settle for 12 h. pNF-κB-luciferase plasmid (100 ng) or control luciferase plasmid plus 10 ng pRLL-TK Renilla plasmid (Promega) was transfected into CCA cells, using Lipofectamine 2000 reagent (Invitrogen). Medium was replaced after 6 h. Luciferase and Renilla signals were measured 48 h after transfection, using the Dual luciferase Reporter Assay kit (Promega) according to a protocol by the manufacturer.

### Apoptosis assay

Human HuCCT-1, TFK-1 and HuH28 CCA cells were plated at a density of 1 × 10^5^ cells/well in six-well plates. After treatment with EF24 (2 μmol/L or 4 μmol/L) or DMSO for 48 h, both detached and attached cells were collected in flow cytometry tubes and centrifuged at 1000 rpm for 5 min to obtain a cell pellet. Cell apoptosis assays were performed as described previously^20^.

### Cell cycle analysis

HuCCT-1, TFK-1 and HuH28 CCA cells were plated at a density of 2 × 10^5^cells/well in six-well plates. After treatment with EF24 (2 μmol/L or 4 μmol/L) or DMSO for 48 hours, both floating and attached cells were collected into flow cytometry tubes and centrifuged at 1,000 rpm for 5 min to obtain a cell pellet. Cell cycle analysis was performed with a Becton Dickinson FACScan. Results were analyzed with ModFit LT software (Verity Software House).

### Real-time reverse transcription-PCR analysis

HuCCT-1, TFK-1 and HuH28 were treated with 2 μmol/L or 4μmol/L of EF24 for 24 h, and then total mRNA was isolated from cells using TRIZOL (Invitrogen, Carlsbad, CA) reagent according to the manufacturer’s protocol. All real-time RT-PCR reactions were performed in duplicate in a 20 μl mixture containing 1 × IQ SYBR Green supermix, 0.2 μM of each primer and 2 μl of cDNA templates. The cDNA generated was utilized for real-time PCR using Jump Start Taq DNA polymerase (Sigma Chemical) and SYBR green nucleic acid stain (Molecular Probes). Primers used in this study were as follows: XIAP 5′-ACGAATGGGGTTCAGTTTCAAGG-3′ and 5′-TTAGCTGCTCTTCAGTACTAATCT-3′. GAPDH: 5′-GTGTCCCCACTGCCAACGTGTCAGT-3′and 5′-GGTGGAGGAGTGGGTGTCGCTGTTGAAGT-3′. The relative expression levels of XIAP were normalized to the reference GAPDH gene.

### Chromatin-immunoprecipitation (ChIP)

ChIP was performed in a similar process as previously reported^44^. Briefly, Human HuCCT-1 CCA cells were plated at a density of 1 × 10^6^ cells/well in 100 mm dishes. After treatment with EF24 (2 μmol/L) or DMSO for 48 h, both detached and attached cells were collected. For each ChIP, 2 μg antibody for NF-κB/p65 or 2 μg nonspecific immunoglobulin G (Santa Cruz) was added and incubated overnight at 4 °C. Amplification of DNA from NF-κB ChIP was carried out with PCR reaction. Primers for XIAP ChIP: sense, 5′-TGTCCTTGTGCCTTTCTTCC-3′; anti-sense, 5′-CCCAGCTCCAGTGTTTCTTC-3′.

### Electrophoretic mobility shift assay (EMSA)

The detailed methodology has been described previously^45^. Briefly, nuclear extract (10 μg) was incubated with 1 μg of poly (deoxyinosinic -deoxycytidylic acid) in binding buffer for 30 min at 4 °C. DNA binding activity was confirmed with a biotin-labeled oligonucleotide bio-NF-κB probe (5′-AGTTGAGGGGACTTTCCCAGGC-3′) using an EMSA kit.

### XIAP shRNA and overexpression

Lentiviral-mediated shRNA knockdown and lentiviral-mediated overexpression of XIAP were performed using lentiviral plasmids (Shanghai Gene Chem Co., Ltd., China). The oligonucleotides containing the XIAP target sequence were: shRNA-1: 5′-TAGGTGAAGGTGATAAAGTAA-3′; shRNA-2: 5′-CACTTGAGGAGTGTCTGGTAA-3′; shRNA-3: 5′-AAGGAGATACCGTGCGGTGCT-3′; shRNA-4: 5′-AAGTGGTAGTCCTGTTTCAGC-3′. One 100 mm dish of HuCCT-1, TFK-1 and HuH28 cells was co-transfected with 3 μg of the plasmids plus 3 μg of each of the packaging vectors (Invitrogen). The media was changed approximately 16 h after transfection, and the cells were cultured for an additional 48–72 h. Experimental cells were incubated with the virus-containing medium overnight in 6-well plates, the media was changed, and the cells were incubated for 24 h. Cells were transferred to 100 mm dishes and infected cells were selected by incubation with EF24.

### Western blot analysis

Standard Western blot assays were used to analyze protein expression, as described^20^. Primary antibodies(1:1000) used are as follows: XIAP, caspase-3, cleaved caspase-3, caspase-9, cleaved caspase-9, caspase-7, caspase-8, PARP, cleaved PARP, cyclin B1, survivin, cdc2, NF-κB, GAPDH and β-actin were purchased from Cell Signaling Technology (Danvers, MA, USA). Antibodies against E-cadherin, N-cadherin and vimentin were purchased from Abcam (Cambridge, MA) (Supplementary Table 1). The secondary antibodies (1:2000), anti-mouse IgG-HRP and anti-rabbit IgG-HRP were also purchased from Santa Cruz Biotechnology (Santa Cruz, CA).

### Animals Studies

Five-week-old male athymic nude mice purchased from Shanghai Laboratory Animal Center were maintained with water and standard mouse chow. All experiments were performed in accordance with the national guidelines and as recommended by the institute’s animal ethics committee. All possible efforts were made to minimize the animals’ suffering and to reduce the number of animal used. All protocols for animal studies were reviewed and approved by the Committee on the Use of Live Animals in Teaching and Research of the Harbin Medical University, Harbin, China. HuCCT-1 CCA cells with XIAP shRNA or overexpression transfection were transplanted into the left and right flank and allowed to form xenograft prior to the initiation of treatments. For the treatment group, EF24 was dissolved in sodium chloride containing 1% dimethyl sulfoxide and injected i.p. at 20 mg/kg for 21 days. The mice in both the treatment and control groups (n = 6 in each group) were sacrificed, and snap-frozen paraffin-embedded tumor tissue blocks were obtained for further analysis.

### *In vivo* Spontaneous Metastasis Assay

Male nude mice (BALB/c) were used in the experiments (n = 10/group). HuCCT-1 cells (3 × 10^6^ cells in 200 μL) were injected into the intraperitoneal cavity as previously described^46^. For the treatment group, EF24 was dissolved in sodium chloride containing 1% dimethyl sulfoxide and injected i.p. at 20 mg/kg for 35 days. The mice in both the treatment and control groups (n = 10 in each group) were sacrificed, and snap-frozen paraffin-embedded tumor tissue blocks were obtained for further analysis.

### Immunohistochemical (IHC) analysis

Expression of XIAP, Ki-67, Cleaved-caspase-3 and -9 (Supplementary Table 1) in tumor tissues was evaluated using the IHC method as described previously^19^,^20^.

### Statistical analysis

All data were expressed as mean ± SD of three independent experiments. Statistical significance was determined using Student’s t-test or ANOVA. A *P* value of less than 0.05 was considered statistically significant. Detailed description of Methods can be found in the online Supporting Information.

## Additional Information

**How to cite this article**: Yin, D.-l. *et al.* EF24 inhibits tumor growth and metastasis via suppressing NF-kappaB dependent pathways in human cholangiocarcinoma. *Sci. Rep.* **6**, 32167; doi: 10.1038/srep32167 (2016).

## Supplementary Material

Supplementary Information
