# Supplementary material for: EF24 inhibits tumor growth and metastasis via suppressing NF-kappaB dependent pathways in human cholangiocarcinoma
Source: Sci Rep. 2016 Aug 30;6:32167. doi: 10.1038/srep32167 (PMC5004153; doi:10.1038/srep32167)
Supplement: Supplementary Information [file srep32167-s1.doc]

**EF24 inhibits tumor growth and metastasis via suppressing NF-kappaB dependent pathways in human cholangiocarcinoma**

Da-long Yin^1,4†^, Ying-jian Liang^1†^, Tong-sen Zheng^1†^, Rui-peng Song^1,4†^, Jia-bei Wang^1^ ,Bo-shi Sun^1^, Shangha Pan^1^, Lian-dong Qu^2^, Jiaren Liu^3^, Hong-chi Jiang^1^*, Lian-xin Liu^1,4^*

**Addresses of authors:** 1 Department of General Surgery, the First Affiliated Hospital of Harbin Medical University; Key Laboratory of Hepatosplenic Surgery, Ministry of Education. No23, Youzheng Street, Nangang District, Harbin, Heilongjiang Province, 150001, P.R.China. China. 2 National Key Laboratory of Veterinary Biotechnology, Harbin Veterinary Research Institute of Chinese Academy of Agricultural Sciences, Harbin, P.R. China. 3 Department of Anaesthesia, Harvard Medical School, Boston, MA, USA. 4 Department of Pharmacology, The State-Province Key Laboratories of Biomedicine- Pharmaceutics of China, Harbin Medical University, Harbin, Heilongjiang 150081, PR China. FAX: +86-451-85553886 TEL: +86-13845159888

*Corresponding author: Lian-xin Liu: LiuLX@ems.hrbmu.edu.cn

Hong-chi Jiang: jianghc@vip.163.com

†These authors contribute equally to this work.

**Supplemental Data:**

**
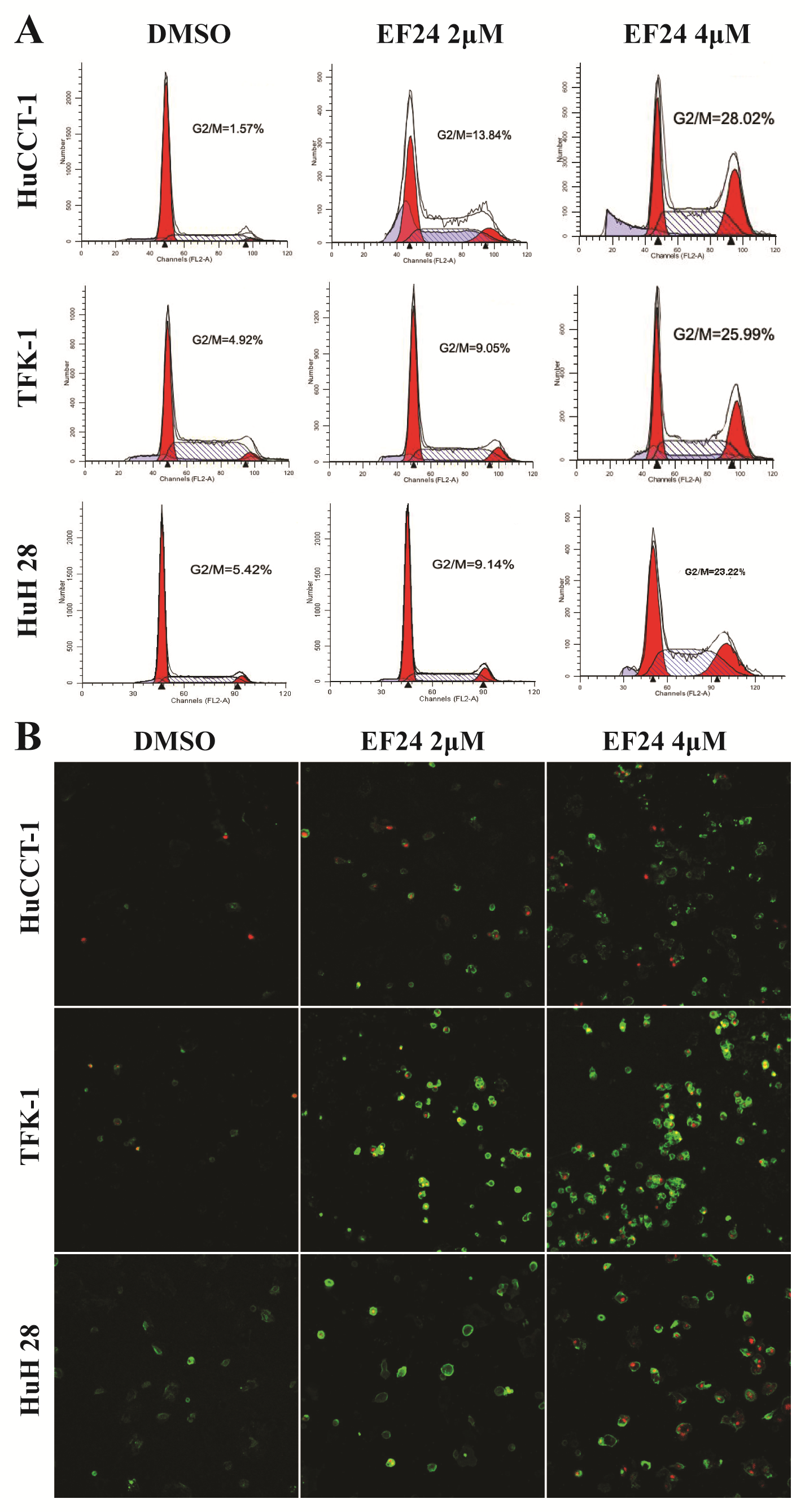
**

**Supplementary Fig S1** (A)Representative images of the cell cycle analysis for HuCCT-1, TFK-1 and HuH 28 after treated with DMSO, EF24 2uM and EF24 4uM. (B) Representative images of confocal microscopy for apoptosis assays.


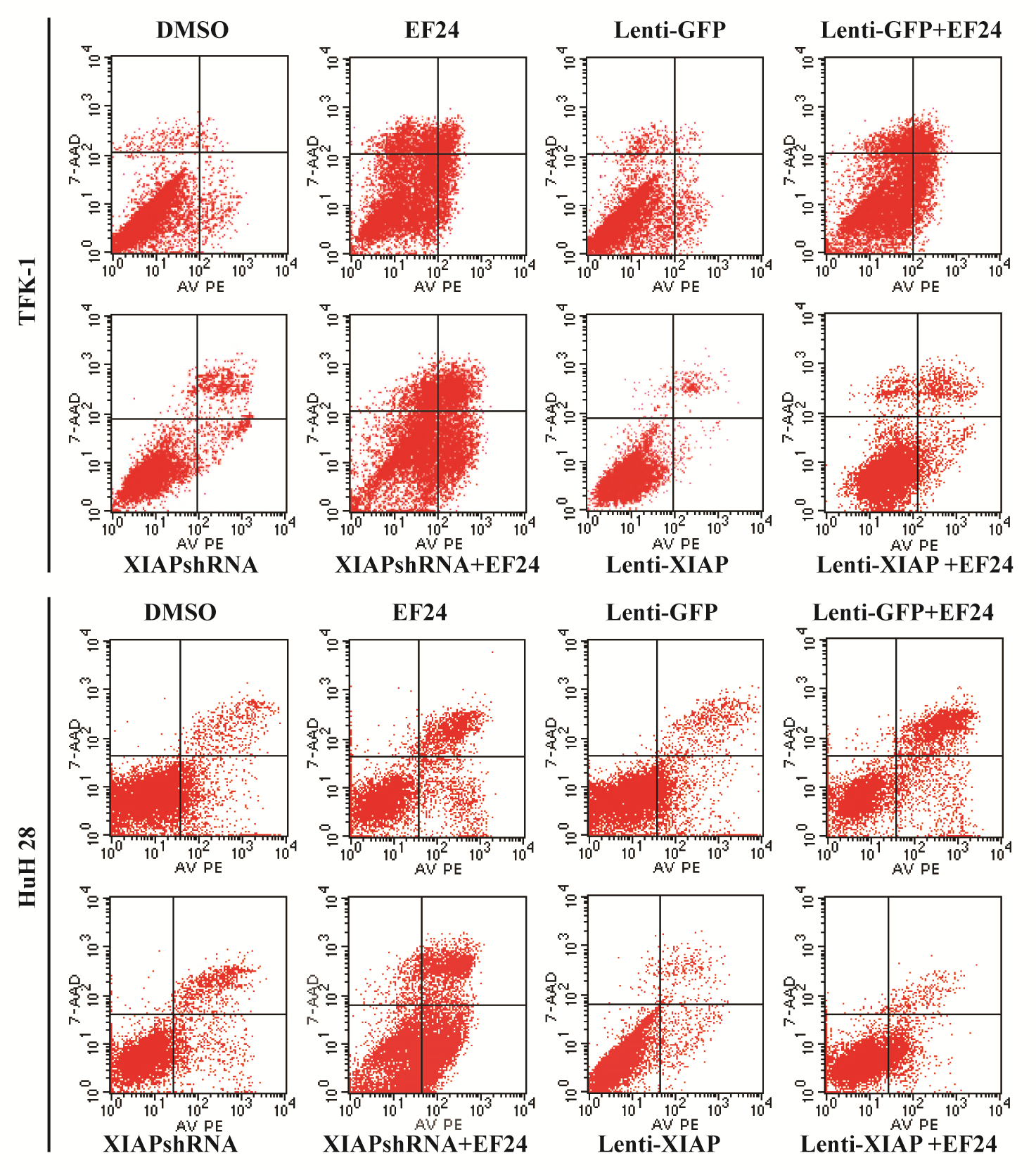


**Supplementary Fig S2** Representative images of the apoptosis assays for TFK-1 and HuH 28 after treated with DMSO, EF24 2uM and EF24 4uM.


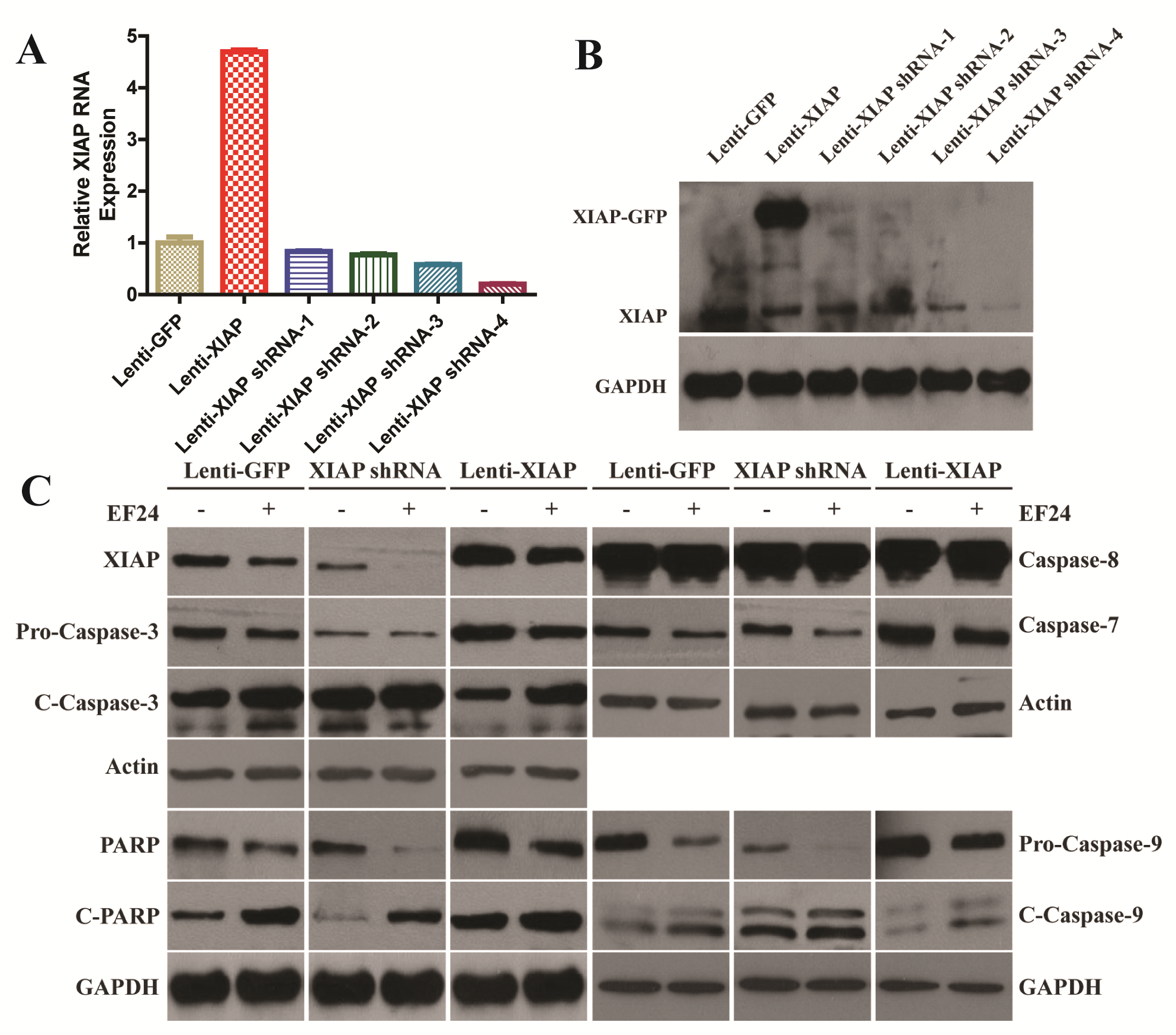


**Supplementary Fig S3** (A) The expression of XIAP was lower after knockdown with LV-shRNA than the control sequences, and higher after transfected with LV-XIAP than the control sequences. (B) Downregulation and upregulation of XIAP protein expression was confirmed by Western blotting. (C) Expression of XIAP, Caspase-8, Caspase-9, Caspase-3, Caspase-7 and PARP were determined by Western blot in HuCCT-1 with Lenti-GFP, Lenti-XIAP shRNA and Lenti-XIAP transfection and EF24 treatment.


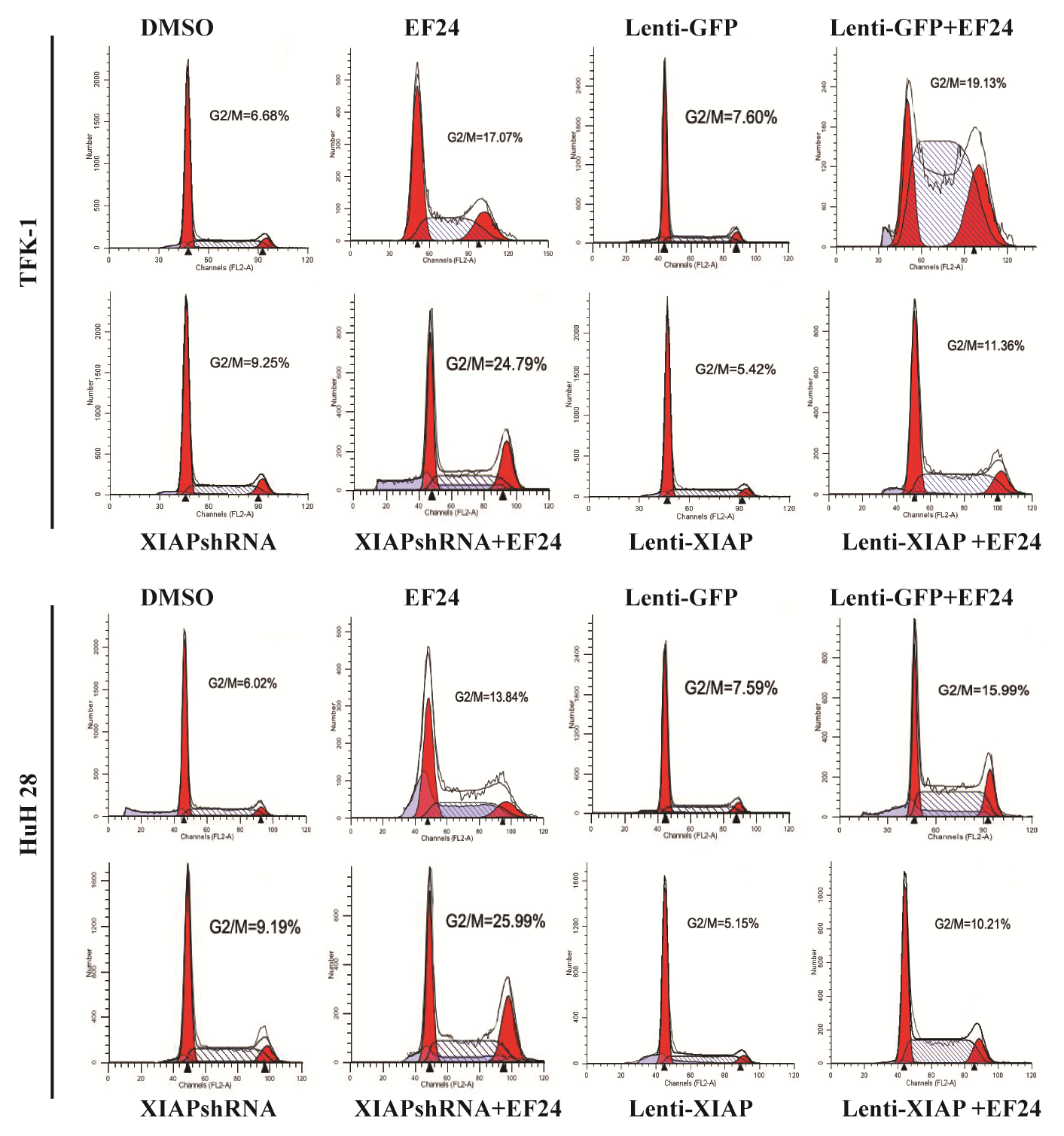


**Supplementary Fig S4** Cell cycle analysis for TFK-1 and HuH 28 with XIAP-shRNA, Lenti-XIAP transfection and EF24 treatment.


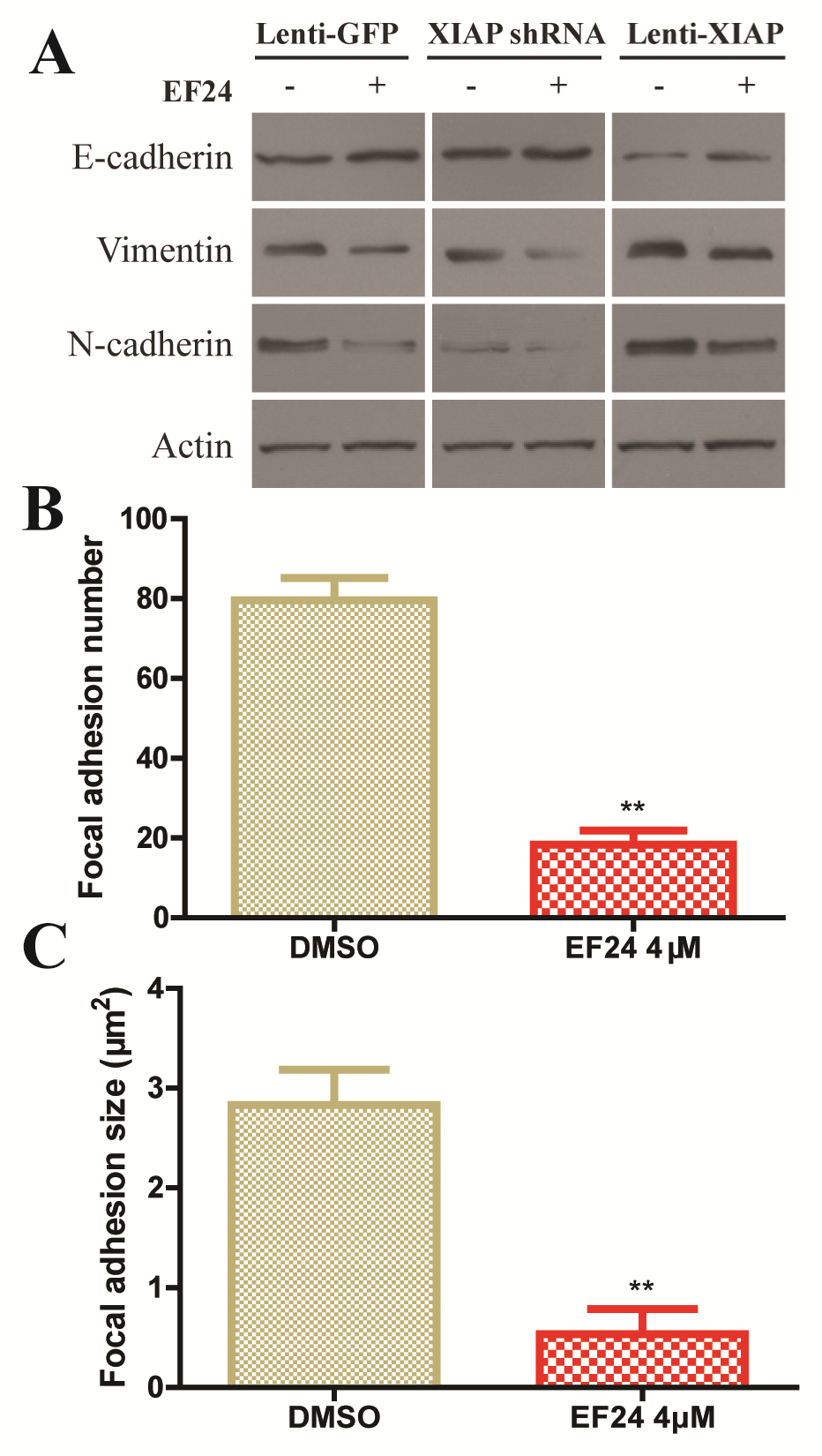


**Supplementary Fig S5** (A) Expression of E-cadherin, Vimentin and N-cadherin were determined by Western blot in HuCCT-1 with Lenti-GFP, Lenti-XIAP shRNA and Lenti-XIAP transfection and EF24 treatment. (B and C) Quantiﬁcation of the average number and size (μm^2^) of Vinculin-containing focal adhesions in HuCCT-1 cells using Image J software. 12 cells were analyzed per condition in each experiment. Data are expressed as mean±SEM of three independent experiments. *p<0.05, **p<0.01 compared with DMSO.


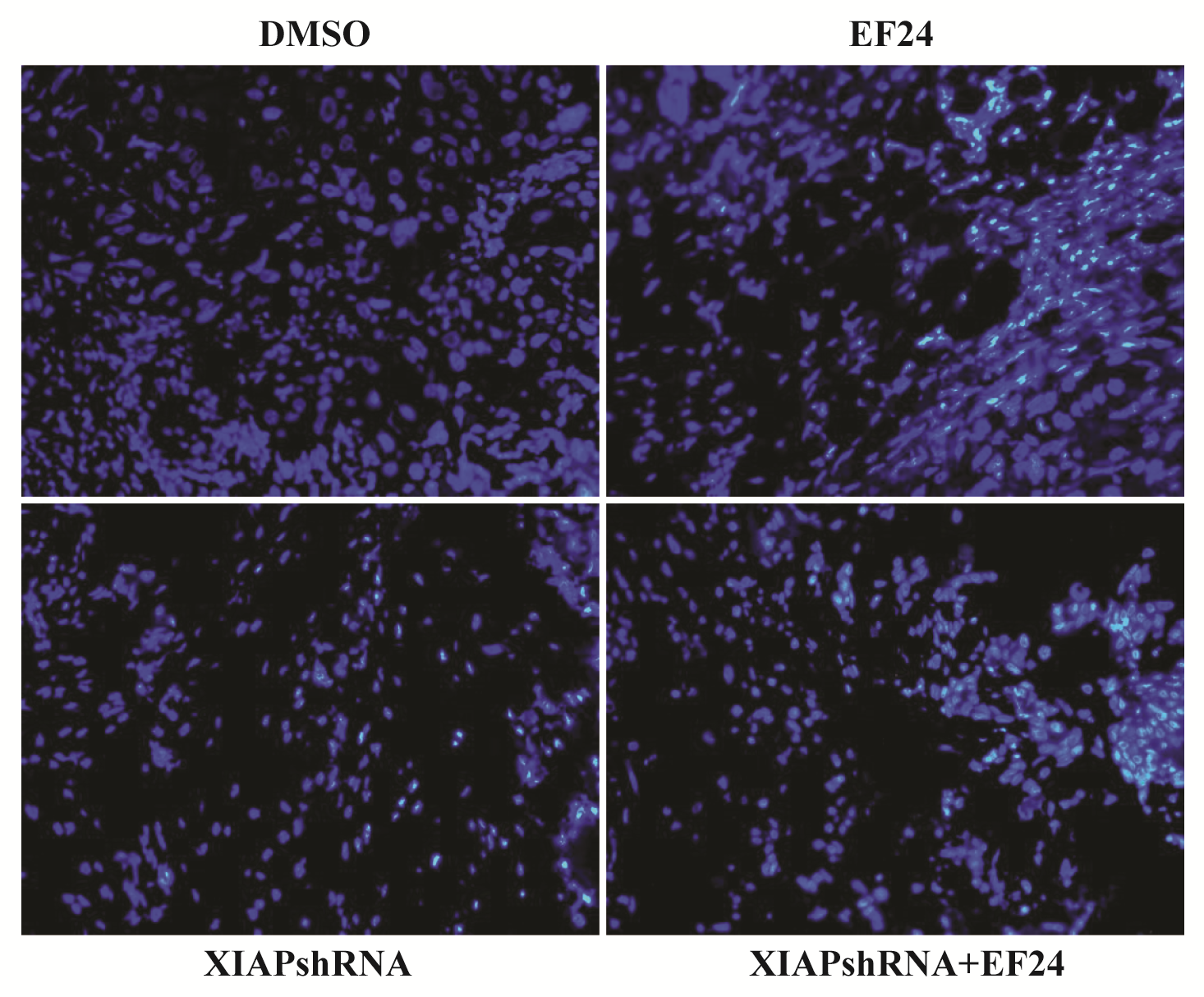


**Supplementary Fig S6** Representative images of TUNEL assays for tumor samples from nude mice after EF24 treatment.

Supplementary Table 1

| **Western blot** | | | | |
| --- | --- | --- | --- | --- |
| **antibody** | **company** | **clone** | **dilution** | |
| XIAP | Cell Signaling Technology | Rabbit anti Human Monoclonal | 1:1000 | |
| P65 | Cell Signaling Technology | Rabbit anti Human Monoclonal | 1:1000 | |
| caspase-3 | Cell Signaling Technology | Rabbit anti Human Monoclonal | 1:1000 | |
| cleaved caspase-3 | Cell Signaling Technology | Rabbit anti Human Monoclonal | 1:1000 | |
| caspase-9 | Cell Signaling Technology | Rabbit anti Human Monoclonal | 1:1000 | |
| cleaved caspase-9 | Cell Signaling Technology | Rabbit anti Human Monoclonal | 1:1000 | |
| caspase-7 | Cell Signaling Technology | Mouse anti Human Monoclonal | 1:1000 | |
| caspase-8 | Cell Signaling Technology | Mouse anti Human Monoclonal | 1:1000 | |
| PARP | Cell Signaling Technology | Rabbit anti Human Monoclonal | 1:1000 | |
| cleaved PARP | Cell Signaling Technology | Rabbit anti Human Monoclonal | 1:1000 | |
| cyclin B1 | Cell Signaling Technology | Mouse anti Human Monoclonal | 1:1000 | |
| cdc2 | Cell Signaling Technology | Mouse anti Human Monoclonal | 1:1000 | |
| E-cadherin | Abcam | Rabbit anti Human Monoclonal | 1:1000 | |
| N-cadherin | Abcam | Rabbit anti Human Monoclonal | 1:1000 | |
| vimentin | Abcam | Mouse anti Human Monoclonal | 1:1000 | |
| Histone H3 | Cell Signaling Technology | Mouse anti Human Monoclonal | 1:1000 | |
| GAPDH | Cell Signaling Technology | Rabbit anti Human Monoclonal | 1:2000 | |
| β-actin | Cell Signaling Technology | Mouse anti Human Monoclonal | 1:2000 | |
| **Immunohistochemical** | | | | |
| **antibody** | **company** | **clone** | | **dilution** |
| XIAP | Cell Signaling Technology | Rabbit anti Human Monoclonal | | 1:100 |
| Ki-67 | Cell Signaling Technology | Mouse anti Human Monoclonal | | 1:100 |
| cleaved caspase-3 | Cell Signaling Technology | Rabbit anti Human Monoclonal | | 1:100 |
| cleaved caspase-9 | Cell Signaling Technology | Rabbit anti Human Monoclonal | | 1:100 |
| **Immunofluorescence** | | | | |
| **antibody** | **company** | **clone** | | **dilution** |
| N-cadherin | Abcam | Rabbit anti Human Monoclonal | | 1:100 |
| vimentin | Abcam | Mouse anti Human Monoclonal | | 1:100 |
| vinculin | Abcam | Mouse anti Human Monoclonal | | 1:100 |

**Supplemental Experimental Procedures:**

**Confocal Microscopy**

Human HuCCT-1, TFK-1 and HuH28 CCA cells were plated at a density of 1×10^4^ cells/well in 12-well plates. After treatment with EF24 (2 μmol/L or 4 μmol/L) or DMSO for 24, 48 or 72 h, 20 μL of the cell suspension was mixed with 15 μL of Annexin V-FITC and 15 μL of Propidium Iodide (BD Biosciences, San Jose, CA, USA), according to the manufacturer’s protocol. Images were captured with a Zeiss laser-scanning confocal microscope (LSM, College of Natural Resources Biological Imaging Facility, UC Berkeley, Berkeley, CA). All images were processed using Photoshop CS4 Suites and Zeiss operating software.

### Immunocytochemistry and TUNEL staining

Cells were seeded on cover slips and fixed with 2% paraformaldehyde in PBS for 20 min at 37°C and then permeabilized with 0.1% Triton X-100 for 5 min at room temperature. Non-specific sites were blocked with 3% BSA for 1 h at 37°C. Cells were incubated with primary antibody (Ki-67, XIAP, cleaved caspase-3 and -9) at 4°C for overnight, washed five times with PBS, and incubated with the corresponding secondary antibodies for 1 h at room temperature. Results were expressed as percentage of Ki-67 ±SE at 40x magnification. A total of 10 fields were examined and counted from three tumors from each treatment group. Apoptotic cells were detected by terminal deoxynucleotidyl transferase–mediating dUTP nick end labeling (TUNEL) staining following the protocol provided by the commercial kit (Roche Diagnostics, Mannheim, Germany). Apoptosis was evaluated by counting TUNEL-positive cells (brown-stained) as well as the total number of cells in five randomly selected fields in each sample.
